# A Prospective Study of Fatty Liver Index and Incident Hypertension: The KoGES-ARIRANG Study

**DOI:** 10.1371/journal.pone.0143560

**Published:** 2015-11-30

**Authors:** Ji Hye Huh, Song Vogue Ahn, Sang Baek Koh, Eunhee Choi, Jang Young Kim, Ki-Chul Sung, Eung Ju Kim, Jeong Bae Park

**Affiliations:** 1 Division of Endocrinology and Metabolism, Department of Internal Medicine, Wonju College of Medicine, Wonju, Korea; 2 Department of Preventive Medicine, Institute of Genomic Cohort, Wonju College of Medicine, Wonju, Korea; 3 Institute of Life Style Medicine, Wonju College of Medicine, Wonju, Korea; 4 Division of Cardiology, Department of Internal Medicine, Yonsei University, Wonju College of Medicine, Wonju, Korea; 5 Division of Cardiology, Department of Medicine, Kangbuk Samsung Hospital, Sungkyunkwan University School of Medicine, Seoul, Korea; 6 Division of Cardiology, Department of Medicine, Korea University Guro Hospital, Korea University College of Medicine, Seoul, Korea; 7 Division of Cardiology, Department of Medicine, Cheil General Hospital, Kwandong University College of Medicine, Seoul, Korea; The Chinese University of Hong Kong, HONG KONG

## Abstract

**Background:**

Although non-alcoholic fatty liver disease is the hepatic manifestation of metabolic syndrome, its influence on hypertension development is poorly understood. We investigated whether fatty liver disease, as assessed by the fatty liver index, could predict the development of hypertension independently of systemic insulin resistance, inflammatory status and adipokine levels.

**Methods:**

Prospective cohort study of 1,521 adults (484 men and 1037 women) aged 40 to 70 years without baseline hypertension examined. An equation was used to calculate fatty liver index and classify patients as follows: fatty liver index <30, no non-alcoholic fatty liver disease; fatty liver index ≥60, non-alcoholic fatty liver disease; and 30≤ fatty liver index <60, intermediate fatty liver index.

**Results:**

During an average of 2.6 years of follow-up, 153 subjects (10.06%) developed hypertension. Fatty liver index was positively associated with baseline blood pressure, homeostasis model assessment of insulin resistance, urinary albumin/creatinine excretion, and high sensitivity C-reactive protein. After adjustment for confounding factors, including markers of insulin resistance, systemic inflammation and adiponectin levels, the odds ratio [95% confidence interval] for the incident hypertension increased in a graded manner with fatty liver index (<30 vs. 30–59 vs. ≥60 = 1 vs. 1.83 [1.16~2.88] vs. 2.09 [1.08~4.055], respectively).

**Conclusions:**

Non-alcoholic fatty liver disease assessed by fatty liver index was an independent risk factor for hypertension. Our findings suggest that fatty liver index, a simple surrogate indicator of fatty liver disease, might be useful for identifying subjects at high risk for incident hypertension in clinical practice.

## Introduction

Hypertension is a growing public health problem because it is closely associated with the risk for chronic kidney disease and morbidity or mortality of cerebral and cardiovascular disease, which demand substantial healthcare costs [1, 2]. Furthermore, the prevalence of hypertension has increased and remains high, as approximately 35% of individuals in East Asia are hypertensive despite significant global and regional efforts [3]. Therefore, it is important to identify adults who are at higher risk for incident hypertension. However, there is still no useful index that predicts incident hypertension.

Non-alcoholic fatty liver disease (NAFLD) is the most common metabolic liver disease, with a prevalence as high as 30% in developed countries [4]. NAFLD is the hepatic component of metabolic syndrome and is also a risk factor for various metabolic disorders including type 2 diabetes, insulin resistance, dyslipidemia, and cardiovascular events [5, 6]. NAFLD and cardiovascular disease may share common pathogeneses such as insulin resistance, aging, and obesity. In addition, altered levels of hepatokines such as fetuin-A, fibroblast growth factor 21, and selenoprotein P in NAFLD might directly affect the progression of atherosclerosis by modulating endothelial dysfunction and infiltration of inflammatory cells into vessel walls. Also, several cross-sectional studies have demonstrated that approximately 50% of subjects with hypertension are known to have NAFLD [7, 8]. However, few studies have examined whether NAFLD itself is associated with the development of hypertension in a community-dwelling cohort, independent of traditional cardiovascular disease risk factors.

Although the gold standard for NAFLD diagnosis is a liver biopsy, this test has limited diagnostic value in a population-based study due to its invasive nature [9]. Therefore, abdominal ultrasonography is the most common technique used to assess the presence of NAFLD in clinical settings. However, abdominal ultrasonography is also expensive and laborious for study participants, and is a subjective operator-dependent examination [10, 11]. Recently, Bedogni et al. developed a simple scoring system called the fatty liver index (FLI) as a predictor of fatty liver diseases [12]. Previous studies have shown that NAFLD assessed by FLI was well-correlated with hepatic steatosis using abdominal ultrasonography in a general population that included Korean patients [13, 14]. In addition, several studies reported that the FLI, as a surrogate indicator of hepatic steatosis, is associated with the development of type 2 diabetes in Asian populations [15]. However, no study has investigated the validity of the FLI for predicting incident hypertension in the general population. We aimed to determine whether NAFLD, as measured by FLI, was associated with new-onset hypertension in a relatively healthy Korean rural population. We also investigated whether fatty liver disease as assessed by FLI was an independent contributor to incident hypertension, regardless of confounding factors such as diabetes, insulin resistance, systemic inflammation status and adipokine levels.

## Materials and Methods

### Study population and design

**Fig 1:**
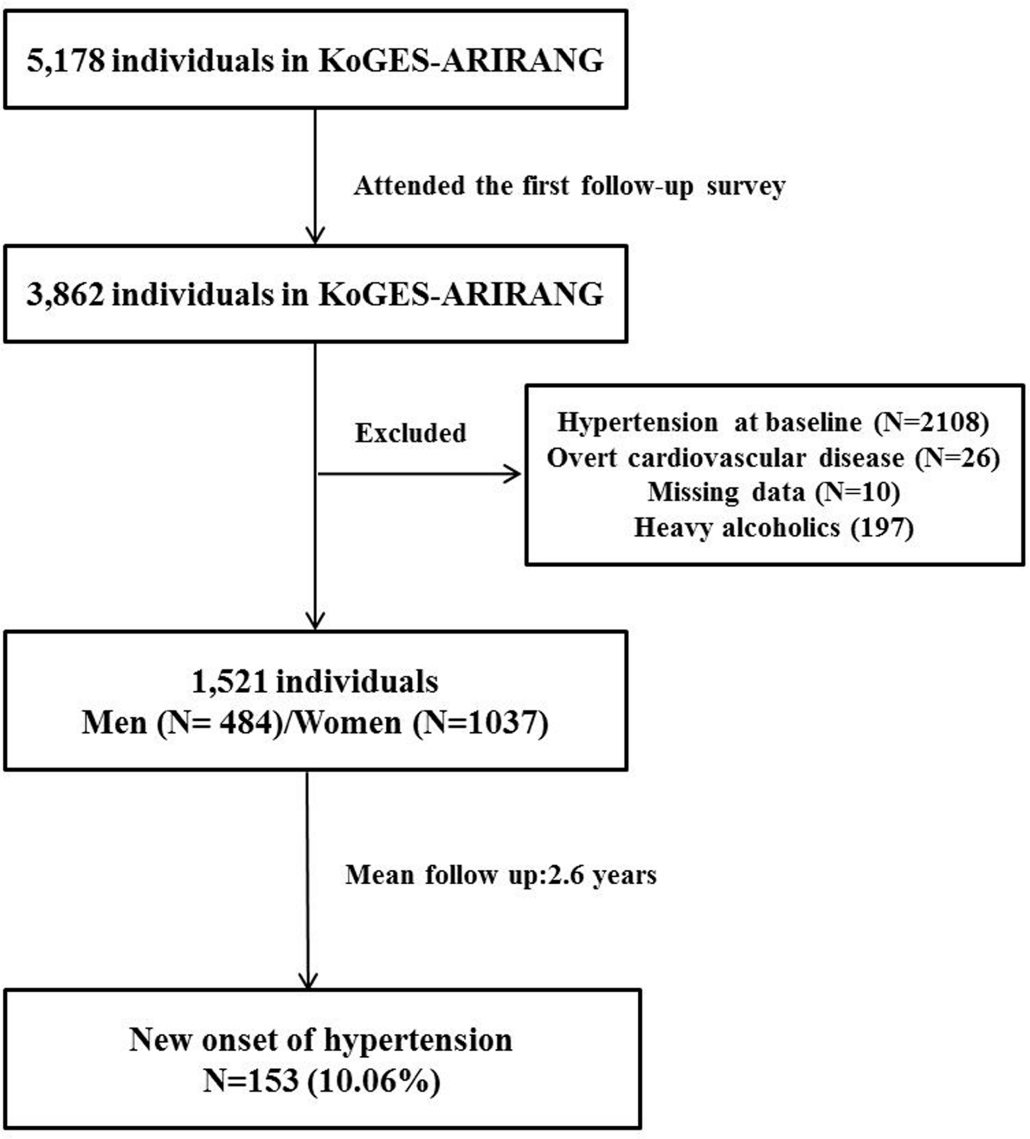
Description of the study populations.

We used data from the Korean Genome and Epidemiology Study on Atherosclerosis Risk of Rural Areas in the Korean General Population (KoGES-ARIRANG), a population-based prospective cohort study, to assess the prevalence, incidence, and risk factors for chronic degenerative disorders such as hypertension, diabetes, metabolic syndrome, and cardiovascular disease [16, 17]. KoGES-ARIRANG invited all adults in rural areas of Wonju and Pyengchang in South Korea, where demographic shifts are infrequent and the population can be followed long term, to participate in the study. The baseline survey, carried out from November 2005 to January 2008, included 5178 adults (2127 men and 3051 women) aged 40–70 years. All study participants were invited to the first follow-up survey (April 2008 to January 2011) and 3862 (74.6%) attended. Subjects with missing data for the FLI (N = 8) and hypertension (N = 2) were excluded. We also excluded subjects who had a history of hypertension (N = 2108) or overt cardiovascular disease (N = 26) at baseline or excessive alcohol intake (alcohol consumption >140 g/week for men and 70 g/week for women) (N = 197). Finally, 1521 participants (484 men and 1037 women) were included in the analysis (Fig 1).

### Ethics Statement

Because the KoGES-ARIRANG study data are publicly available, ethical approval was not required for this study. Prior to the survey, all participants were informed that they had been randomly chosen to participate in the KoGES-ARIRANG survey with the right to refuse to be involved in further analyses, and signed informed consents were obtained. We received the data in fully anonymized form. The study was carried out in accordance with the ethical standards of the Helsinki Declaration.

### Data collection

At baseline and at the follow-up examination, study participants completed a standardized medical history and lifestyle questionnaire and underwent a comprehensive health examination according to standard procedures. Body weight and height were measured while participants were wearing light indoor clothing without shoes. Blood pressure was measured in the right arm using a standard mercury sphygmomanometer after the participant had rested for at least 5 min in a quiet room (Baumanometer, Copiague, NY, USA). With participants seated, an appropriately-sized cuff was applied snugly around the upper right arm at the heart level. The appropriate cuff size was chosen for each subject according to mid-arm circumference. Two measurements were made with at least 5 min intervals in between, and the mean of the two measurements was used in the analyses. According to the Eighth Joint National Committee (JNC-8) guidelines [18], hypertension was defined as a systolic blood pressure of at least 140 mmHg or a diastolic blood pressure of at least 90 mmHg, or current use of antihypertensive agents. Diabetes mellitus was defined as fasting serum glucose of at least 126 mg/dL or current use of blood glucose–lowering agents at baseline. Baseline information on smoking status and current alcohol intake was collected with a self-reported questionnaire (yes/no). Subjects who answered yes to the question: ‘‘Do you perform physical exercise regularly enough to make you sweat?” were assigned to the regular exercise group. A venous blood sample was drawn from study participants after fasting for ≥12 h or overnight. The serum concentrations of adiponectin were measured by radioimmunoassay (RIA) (LINCO Research, Inc., USA) with intra-assay and inter-assay coefficients of variation ranging from 2.9% to 6.6% for adiponectin. Fasting glucose was determined by a glucose oxidase-based assay and fasting insulin was determined by a double-antibody radioimmunoassay (Biosource Europe SA, Nivelles, Belgium). Serum concentrations of total cholesterol, low density lipoprotein (LDL) cholesterol, high density lipoprotein (HDL) cholesterol, triglycerides (TG), aspartate aminotransaminases (AST), alanine aminotransaminases (ALT), and γ-Glutamyltransferase (GGT) were determined by enzymatic methods (Advia 1650, Siemens, Tarrytown, NY, USA). High sensitivity C-reactive protein (hsCRP) was measured by the Denka Seiken (Tokyo, Japan) assay, which has been validated against the Dade Behring method. Homeostasis model assessment of insulin resistance (HOMA-IR) values were calculated using the following formula: fasting plasma glucose (milligrams per deciliter) ×fasting insulin (milliinternational units per milliliter))/22.5 [19].

### Definition of hepatic steatosis according to FLI

The FLI was used as a surrogate measure for fatty liver. This index was calculated according to a previously published report by Bedogni et al. [12]: FLI = [e ^0.953^×log_e_ (TG) + 0.139×BMI+0.718×log_e_ (GGT) +0.053×waist circumference–15.745)] / [1+e^0.953^× log_e_ (TG) + 0.139×BMI+0.718×log_e_ (GGT) + 0.053×waist circumference–15.745] × 100, with triglycerides measured in mmol/l, GGT in U/l, and waist circumference in cm. The score ranges from 0 to 100. According to the previous study by Bedogni et al., FLI <30 can be used to rule out (sensitivity = 87%; negative likelihood ratio = 0.2) and FLI ≥60 to rule in hepatic steatosis (specificity = 86%; positive likelihood ratio = 4.3). Thus, we classified participants into three groups according to the value of FLI: FLI <30 was defined as not having NAFLD, and FLI ≥60 was defined as having NAFLD. Patients with FLI 30–59 were defined as having intermediate FLI.

### Statistical analyses

Data are expressed according to the properties of the variable. Continuous variables are presented as means and standard deviations. Categorical variables are presented as frequencies and percentages. In order to compare incident hypertension and FLI categories, we performed the two-sample t-test, one-way analysis of variance (ANOVA), and chi-square test (Fisher’s exact test), as appropriate. The association between the FLI and baseline metabolic parameters was evaluated using Pearson correlation analysis. Multivariate logistic regression was used to assess the independent association of baseline FLI with incident hypertension. We used three models with progressive degrees of adjustment. First, we performed an age (continuous variable) and sex adjusted analysis. Second, we further adjusted for baseline systolic and diastolic blood pressure (continuous variable), smoking (yes/no), alcohol intake (yes/no), regular exercise (yes/no) and diabetes. Finally, we further adjusted for baseline levels of hs-CPR (continuous variable), HOMA-IR (log-transformed continuous variable), ALT (log-transformed continuous variable), serum creatinine (continuous variable) and adiponectin (continuous variable). Results were expressed as odds ratios with 95% confidence intervals (CI). P values less than 0.05 were considered statistically significant and all statistical analyses were performed using SAS 9.2 Ver. (SAS Institute Inc., Cary, NC, USA).

## Results

### Patient characteristics

Baseline clinical and biochemical characteristics of participants based on the development of hypertension during 2.6 years are shown in Table 1. The overall incidence of hypertension during this period was 10.06% (N = 153). Subjects who developed hypertension had significantly higher baseline age, waist circumference, body mass index, blood pressure, total cholesterol, and serum creatinine than those who did not. The AST, ALT, total bilirubin, and GGT levels at baseline were not different between the incident and the non-incident hypertension groups. FLI values were higher in the incident hypertension group (30.95 ± 22.16 vs. 21.69 ± 20.15, P<0.001). The baseline demographic and clinical characteristics of the subjects, who were classified into three groups according to baseline FLI values, are presented in Table 2. 1098 (72.19%), 299 (19.66%), and 124 (8.15%) subjects were classified as not having NAFLD (<30), intermediate FLI (30–59), or having NAFLD (≥60), respectively. As the FLI increased, subjects were more likely to be male, current smokers, obese, and insulin resistant. Subjects with higher FLI also had higher baseline blood pressure, fasting/postprandial plasma glucose, TG, AST, ALT, GGT, and urinary albumin/creatinine excretion. Subjects with higher FLI had lower HDL cholesterol and adiponectin than other groups. Incident hypertension gradually increased across all FLI groups.

**Table 1:** Baseline characteristics according to incident hypertension.

	Incident hypertension (+)	Incident hypertension (-)	
	N = 153 (10.06%)	N = 1368 (89.94%)	P-value
Age (years)	57.56 ± 7.8	53.1 ± 8.07	< .0001
Gender (male)	59 (38.56%)	425 (31.07%)	0.0591
BMI (kg/m^2^)	25.03 ± 2.87	23.7 ± 2.91	< .0001
Waist circumference (cm)	85.45 ± 7.82	80.25 ± 8.47	< .0001
Weight (kg)	62.79 ± 9.46	59.37 ± 9.02	< .0001
Systolic blood pressure (mmHg)	122.9 ± 9.99	116.9 ± 11.07	< .0001
Diastolic blood pressure (mmHg)	75.78 ± 6.53	73.5 ± 7.45	0.0003
HbA1c (%)	5.59 ± 0.71	5.5 ± 0.72	0.1397
Fasting glucose (mg/dl)	93.8 ± 13.12	92.93 ± 19.43	0.4599
HOMA-IR	2.0 ± 1.25	1.9 ± 1.15	0.2783
Total cholesterol (mg/dl)	204 ± 34.32	196.3 ± 35.67	0.0111
HDL- C (mg/dl)	45.37 ± 9.89	46.32 ± 10.52	0.2891
Triglycerides (mg/dl)	136.4 ± 76.28	124.4 ± 80.78	0.0788
LDL-C (mg/dl)	122.9±28.37	115.6±30.75	0.0051
AST (IU/l)	27.15 ± 8.74	26.21 ± 15.15	0.2514
ALT (IU/l)	24.51 ± 13.61	22.51 ± 14.86	0.1124
GGT (IU/l)	24.47 ± 20.2	25.17 ± 54.86	0.7503
Total bilirubin (mg/dl)	0.84 ± 0.28	0.86 ± 0.31	0.456
Adiponectin (μg/ml)	10858.2 ± 5509	10978.9 ± 5321	0.7982
Fatty liver index	30.95 ± 22.16	21.69 ± 20.15	< .0001
Creatinine (mg/dl)	0.93 ± 0.15	0.91 ± 0.14	0.0365
Urine ACR (mg/g)	3.26 ± 9.45	1.89 ± 6.49	0.1194
hsCRP (mg/l)	1.8 ± 3.51	1.64 ± 4.61	0.6043
Alcohol use	64 (42.38%)	452 (33.16%)	0.0233
Current smoker	38 (25.17%)	303 (22.18%)	0.4045
Regular exercise	34 (22.37%)	409 (30.01%)	0.0495

Data are expressed as mean±standard deviation.

BMI, Body mass index; HOMA-IR, homeostasis model assessment of insulin resistance, HDL-C, High density lipoprotein cholesterol; LDL-C, Low density lipoprotein cholesterol; AST, aspartate aminotransferase; ALT, alanine aminotransferase; GGT, gamma-glutamyltransferase; urine ACR, urine albumin/creatinine ratio

**Table 2:** Baseline characteristics according to fatty liver index (FLI) category.

	FLI			
	<30	30~59	≥60	P-value
	N = 1098 (72.19%)	N = 299 (19.66%)	N = 124 (8.15%)	
Age (years)	53.05 ± 8.14	55.35 ± 8.18	53.6 ± 7.55	< .0001
Gender (male)	268 (24.41%)	133 (44.48%)	83 (66.94%)	< .0001
BMI (kg/m^2^)	22.8 ± 2.32	26.05 ± 2.22	27.7 ± 3.11	< .0001
Waist circumference (cm)	77.37 ± 6.65	88.16 ± 5.58	93.19 ± 6.42	< .0001
Weight (kg)	56.59 ± 7.21	65.82 ± 7.66	72.68 ± 8.69	< .0001
Systolic blood pressure (mmHg)	116.69 ± 11.26	120.09 ± 10.65	118.64 ± 9.76	< .0001
Diastolic blood pressure (mmHg)	73.29 ± 7.62	74.81 ± 6.72	75.06 ± 6.44	0.0008
HbA1c (%)	5.42 ± 0.64	5.72 ± 0.88	5.76 ± 0.8	< .0001
Fasting glucose (mg/dl)	91.06 ± 16.02	97.81 ± 26.2	98.75 ± 18.19	< .0001
Postprandial glucose (mg/dl)	79.02 ± 74.81	101.98 ± 92.02	125.44 ± 72.98	< .0001
Fasting insulin (μIU/ml)	7.74 ± 3.42	9.67 ± 5	10.39 5.39	< .0001
HOMA-IR	1.73 ± 0.99	2.29 ± 1.3	2.53 ± 1.68	< .0001
Total cholesterol (mg/dl)	192.32 ± 32.96	210.38 ± 40.01	207.36 ± 36.77	< .0001
HDL- C (mg/dl)	47.66 ± 10.42	42.76 ± 9.52	41.88 ± 9.84	< .0001
Triglycerides (mg/dl)	101.61 ± 44.35	165.98 ± 73.85	240.56 ± 163.37	< .0001
LDL-C (mg/dl)	113.17±28.2	127.3±33.66	117.44±36.67	< .0001
AST (IU/l)	24.42 ± 10.27	28.24 ± 16.92	38.35 ± 28.69	< .0001
ALT (IU/l)	19.42 ± 9.05	27.99 ± 19.33	39.15 ± 24.81	< .0001
GGT (IU/l)	15.95 ± 10.52	31.78 ± 30.07	90.01 ± 160.24	< .0001
Total bilirubin (mg/dl)	0.87 ± 0.31	0.83 ± 0.27	0.84 ± 0.33	0.1523
Adiponectin (μg/ml)	11791 ± 5326	9607 ± 4936	7244 ± 3958	< .0001
Creatinine (mg/dl)	0.89 ± 0.13	0.95 ± 0.15	0.99 ± 0.14	< .0001
Urine ACR (mg/g)	1.53 ± 3.48	2.23 ± 4.76	5.42 ± 18.78	< .0001
hsCRP (mg/l)	1.46 ± 4.79	2.08 ± 3.45	2.32 ± 4.07	0.0255
Alcohol use	327 (29.89%)	109 (36.58%)	80 (65.57%)	< .0001
Current smoker	185 (16.89%)	91 (30.54%)	65 (52.42%)	< .0001
Regular exercise	331 (30.23%)	83 (27.95%)	29 (23.58%)	0.264

Data are expressed as mean±standard deviation.

BMI, Body mass index; HOMA-IR, homeostasis model assessment of insulin resistance, HDL-C, High density lipoprotein cholesterol; LDL-C, Low density lipoprotein cholesterol; AST, aspartate aminotransferase; ALT, alanine aminotransferase; GGT, gamma-glutamyltransferase; urine ACR, urine albumin/creatinine ratio; hsCRP, high-sensitivity C-reactive protein

### Correlation between FLI and metabolic parameters

Table 3 presents correlation analysis between FLI and baseline major metabolic parameters. FLI values were positively correlated with systolic and diastolic blood pressure, HbA1c, fasting/postprandial plasma glucose, HOMA-IR, AST, ALT, total cholesterol, urinary albumin/creatinine ratio, and hsCRP. FLI values were significantly negatively associated with HDL cholesterol and adiponectin.

**Table 3:** Correlation between fatty liver index (FLI) and metabolic parameters.

	Correlation coefficient	p-value
Systolic BP mmHg	0.121	< .0001
Diastolic BP mmHg	0.114	< .0001
HbA1c* (%)	0.242	< .0001
Fasting glucose* (mg/dl)	0.234	< .0001
Postprandial glucose (mg/dl)	0.216	< .0001
Fasting insulin* (μIU/ml)	0.294	< .0001
HOMA-IR*	0.337	< .0001
Total cholesterol (mg/dl)	0.24	< .0001
HDL-C (mg/dl)	-0.265	< .0001
LDL-C (mg/dl)	0.168	< .0001
AST* (IU/l)	0.36	< .0001
ALT* (IU/l)	0.511	< .0001
Urine ACR* (mg/g)	0.202	< .0001
Adiponectin (μg/ml)	-0.315	< .0001
Creatinine (mg/dl)	0.29	< .0001
hs-CRP (mg/l)	0.081	0.0016

Values are Pearson correlation coefficients between variables and FLI

*Log transformed variables

### The risk for incident hypertension according to fatty liver index groups

The risk of incident hypertension according to the baseline FLI groups is shown in Table 4. After adjustment for age and gender, the odds ratios for incident hypertension increased across fatty liver index groups. This trend remained statistically significant even after further adjustment for other confounding variables, including baseline blood pressure, smoking status, regular exercise, alcohol intake, and diabetes, which are established risk factors for hypertension. In full adjusted model including insulin resistance status, liver enzyme, hsCRP, serum creatinine level and adiponectin, the odds ratios (95% CI) for developing hypertension in the 30≤FLI≤59 and FLI ≥60 were 1.87 (1.2–2.91) and 2.22 (1.16–4.25) comparing to those in the FLI<30. We also investigated FLI values at the end of the study and the change of FLI values during follow-up period in participants. As a result, we found that subjects who developed hypertension had still higher FLI values at the end of study than the other group (32.87±22.23 vs. 22.15±19.49,P<0.001). In addition, FLI values were more frequently increased in incident hypertension group than in non-incident hypertension group during follow-up period (41.83% vs. 32.31%, P = 0.0051) (S1 Table).

**Table 4:** Odds ratios (OR) and 95% confidence intervals for hypertension according to fatty liver index (FLI) categories.

	FLI			
	<30	30~60	≥60	P-value
Incident hypertension	84 (7.65%)	48 (16.05%)	21 (16.94%)	< .0001
Crude OR	1	2.31 (1.58~3.38)	2.46 (1.46~4.14)	< .0001
Model 1	1	2.04 (1.38~3.02)	2.47 (1.43~4.26)	0.0001
Model 2	1	1.79 (1.19~2.68)	2.07 (1.15~3.7)	0.0042
Model 3	1	1.83 (1.16~2.88)	2.09 (1.08~4.05)	0.0145

Model 1: Adjusted for age, gender

Model 2: Model 1 + further adjusted for baseline systolic blood pressure, baseline diastolic blood pressure, smoking, regular exercise, alcohol intake, and diabetes

Model 3: Model 2 + further adjusted for log ALT, log HOMA-IR, hsCRP, serum creatinine, adiponectin

## Discussion

In this rural cohort study, we found that NAFLD, as assessed by FLI, was significantly associated with development of hypertension during a 2.6-year period in Korean adults. Furthermore, the FLI was an independent predictor for incident hypertension, regardless of insulin resistance status, as reflected by HOMA-IR and systemic inflammatory status reflected by hsCRP. To the best of our knowledge, this is the first population-based study to reveal an association between fatty liver disease as assessed by FLI and incident hypertension. Our results suggest that fatty liver disease itself independently increases the risk of hypertension and that the FLI may be a useful clinical marker for prediction of incident hypertension.

NAFLD is an emerging risk factor for cardiovascular disease, as demonstrated by the significant association between NAFLD and fatal and nonfatal cardiovascular disease [20, 21]. Aneni et al. reported that NAFLD was associated with pre-hypertension as well as hypertension [22]. Moreover, Lopez et al. also demonstrated that NAFLD is associated with blood pressure in hypertensive and non-hypertensive individuals with normal liver enzyme levels [7]. However, because these were cross-sectional studies, they could not confirm a cause-effect relationship between NAFLD and hypertension. In one study, NAFLD assessed by ultrasonography was associated with incident hypertension in Korean men [23]; however, this study did not measure adipokines such as adiponectin, which could explain the association between fatty liver disease and incident hypertension. In addition, because this study did not consider insulin resistance and inflammatory status when analyzing the association between NAFLD and incident hypertension, they could not clarify the independent effect of NAFLD on incident hypertension. Therefore, we assessed whether NAFLD contributes to the development of hypertension independently of insulin resistance and systemic inflammation in relatively healthy Korean adults.

We observed a significant association between NAFLD as assessed through FLI and the development of hypertension. The observed link between fatty liver disease and hypertension could be explained by multiple mechanisms. First, renin–angiotensin system (RAS) dysregulation in NAFLD might lead to the development of hypertension, as RAS dysregulation may play a key role in hepatic inflammation and fibrosis [24, 25]. RAS inhibition in experimental animal models downregulates pro-inflammatory/pro-fibrotic cytokines, reduces activation of hepatic stellate cells, attenuates oxidative stress, and inhibits hepatic inflammation and fibrosis [26, 27]. We also observed that subjects with NAFLD assessed by FLI had higher urinary albumin excretion, and FLI was positively associated with urinary albumin excretion. These findings suggest that NAFLD is associated with RAS dysregulation, and that this RAS dysregulation may play a substantial role in the development of hypertension. Second, insulin resistance and systemic inflammation in NAFLD may lead to the development of hypertension. Chronic inflammation of the liver, secondary to the storage of free fatty acids in the form of triglycerides, could cause systemic insulin resistance by inducing proinflammatory cytokines via activation of NF-κβ [28, 29] [30]. In addition, insulin resistance induced by chronic systemic inflammation is a known risk factor in the development and the progression of NAFLD. We found that that both HOMA-IR (as a marker of insulin resistance) and hsCRP (as a marker of systemic chronic inflammation) were higher in subjects with NAFLD. Insulin resistance is also an important risk factor for cardiovascular disease, as this results in increased secretion of proinflammatory cytokines, such as tumor necrosis factor-α and interleukin-6 [31]. Moreover, elevated vascular sympathetic activation induced by insulin resistance might result in the development of hypertension [32]. However, in our study, the strong relationship between NAFLD and incident hypertension remained even after adjustment for HOMA-IR and hsCRP. Therefore, we hypothesize that fatty liver disease itself contributes to the development of hypertension, independently of systemic inflammation and insulin resistance. Third, altered secretion of adipokines in fatty liver disease might lead to the development of hypertension. Leptin-mediated sympatho-activation is a well-known mechanism of obesity-induced hypertension [33, 34]. Furthermore, adiponectin also regulates vascular homeostasis by affecting signaling pathways in endothelial cells and modulating inflammatory responses in the subendothelial space [35]. Given the known pathogenic role of leptin/adiponectin in hypertension development, lower adiponectin and higher leptin in subjects with NAFLD may contribute to increase of blood pressure. But, we also observed the association between FLI and incident hypertension remained statistically significant even after adjustment for adiponectin level in our study. Finally, hepatokines from a fatty liver can modulate inflammatory processes that in turn mediate vascular disease [36]. This mechanism might also explain the independent role of NAFLD in the progression of hypertension.

We observed significant effectiveness of FLI (≥30) for predicting incident hypertension. As fatty liver disease is rapidly increasing in prevalence, it is important to identify high-risk subjects early to select subjects who need further imaging study such as ultrasonography and magnetic resonance spectroscopy. Radiological modalities which are widely used for detecting fatty liver disease are rather inaccurate because milder degrees of steatosis (<33% of fat in hepatocytes) cannot be fully detected [37]. However, FLI is a simple and easily accessible index in clinical practice, as it only requires measurements of BMI, waist circumference, GGT, and TG. The FLI can detect fatty liver disease assessed by ultrasonography with considerable accuracy [12] and it has been well validated in a Korean population [13]. Therefore, FLI is simple and useful clinical marker for predicting the presence of hepatic steatosis as well as the risk of metabolic disorders like diabetes and hypertension. Considering that hypertension is the most important risk factor for cardiovascular disease, the FLI also can be applied to predict and prevent further cardiovascular disease.

The major strength of our study is that the data were obtained from a rural cohort study that included a large number of subjects. Moreover, this is the first population-based prospective study that extensively investigated the potential role of hepatic steatosis in the pathogenesis of hypertension. However, this study has some limitations. First, our study used FLI as a surrogate marker for NAFLD, because this cohort study did not include imaging studies such as ultrasounds or magnetic resonance spectroscopy with liver biopsy, which is a gold standard for diagnosing NAFLD. However, other recent studies also showed some important findings using these validated NAFLD predictive models to overcome the limitation of abdominal sonography based diagnosis [38, 39]. Second, because each component of FLI such as waist circumference, BMI, TG, and GGT is a risk factor for hypertension by itself, NAFLD may not be an independent predictor for incident hypertension. Third, because the follow-up period of our cohort was too short (2.6 years) and about 25% of the sample did not complete the follow-up visit, we could not analyze data in a large sample. Fourth, we could not measure hepatokines such as fetuin-A, fibroblast growth factor 21, and selenoprotein P and levels of renin, angiotensin II and aldosterone which could explain the mechanism for an independent role of NAFLD in incident hypertension. Finally, because our study participants were Korean rural adults, it is unclear whether our results could be applied to other regional populations and other ethnic groups.

In conclusion, our study is the largest longitudinal cohort study to examine the association between fatty liver disease and incident hypertension in a relatively healthy rural population. Our results demonstrated that the FLI, a simple surrogate indicator of hepatic steatosis, is a valuable and easily accessible tool for identifying subjects at high risk for hypertension. In addition, fatty liver disease itself contributes to the development of hypertension, independently of systemic insulin resistance, inflammatory status and adiponectin levels. Considering the worldwide increase in fatty liver disease and hypertension, further studies are warranted to elucidate the mechanism for the independent role of fatty liver disease in incident hypertension. In addition, a larger study is needed to validate the FLI for the prediction of incident hypertension.

## Supporting Information

S1 Dataset (XLSX)

S1 Table Fatty liver index changes according to incident hypertension (DOCX)
