# Supplementary material for: A Prospective Study of Fatty Liver Index and Incident Hypertension: The KoGES-ARIRANG Study
Source: PLoS One. 2015 Nov 30;10(11):e0143560. doi: 10.1371/journal.pone.0143560 (PMC4664241; doi:10.1371/journal.pone.0143560)
Supplement: S1 Table — (DOCX) [file pone.0143560.s002.docx]

**S1 Table. Fatty liver index changes according to incident hypertension**

|  | **Incident hypertension (+)** | | **Incident hypertension (-)** |  |
| --- | --- | --- | --- | --- |
|  | **N=153 (10.06%)** | | **N=1368 (89.94%)** | **P-value** |
| Baseline FLI | | 30.95±22.16 | 21.69±20.15 | <.0001 |
| Follow up FLI | | 32.87±22.23 | 22.15±19.49 | <.0001 |
| Changes of FLI | |  |  | 0.0051 |
| Decreased | | 55 (35.95%) | 450 (32.97%) |  |
| No change | | 34 (22.22%) | 474 (34.73%) |  |
| Increased | | 64 (41.83%) | 441 (32.31%) |  |
